# EXAMINING SELF-REPORTED SLEEP QUALITY WITH COGNITIVE PERFORMANCE IN OLDER HISPANIC ADULTS: THE COMPADRE STUDY

**DOI:** 10.1093/geroni/igae098.3520

**Published:** 2024-12-31

**Authors:** Vanessa Young, Carlos Gaona, Luis Serrano-Rubio, Chen-Pin Wang, Tiffany Kautz, Jeffrey Kaye, Zachary Beattie, Mitzi Gonzales

**Affiliations:** The University of Texas Health Science Center San Antonio, San Antonio, Texas, United States; The University of Texas Health Science Center San Antonio, San Antonio, Texas, United States; The University of Texas Health Science Center San Antonio, San Antonio, Texas, United States; The University of Texas Health Science Center San Antonio, San Antonio, Texas, United States; The University of Texas Health Science Center San Antonio, San Antonio, Texas, United States; Oregon Health & Science University, Portland, Oregon, United States; Oregon Health & Science University, Portland, Oregon, United States; Cedars-Sinai Medical Center, Los Angeles, California, United States

## Abstract

Poor sleep quality has been associated with worse cognition performance. As sleep quality is multidimensional, examining separate factors captured by the PROMIS™ Sleep Disturbance 8b scale, insomnia, and sleep dissatisfaction, may provide greater insight into aspects most pertinent to cognitive health. Herein, we evaluated the associations between self-reported sleep quality, dissatisfaction, and insomnia with cognition in older Hispanic adults and examined whether self-reported physical activity modifies these associations. Participants completed self-report measures of sleep quality (PROMIS-Sleep Disturbance 8b) and physical activity (Rapid Assessment of Physical Activity). The National Alzheimer’s Coordinating Center UDSv3 measured cognitive abilities. Associations between self-reported sleep with cognition were examined using linear regression models adjusted for age, sex, and education. Interactions with physical activity were evaluated. A total of 85 participants were included (mean age 69.9[SD 4.8] years; 72% Female; 100% Hispanic). Self-reported sleep quality, dissatisfaction and insomnia were not significantly associated with any cognitive outcomes (p>0.05) and there were no significant interactions with physical activity (p>0.05). Although self-reported sleep quality measures were not associated with cognitive performance in this exclusively Hispanic cohort, previous research using objective sleep measures has shown a connection. This highlights the need for objective assessments that capture various aspects of sleep, such as architecture, microarousals, and sleep-disordered breathing. Digital biomarkers of sleep and physical activity, currently underway in the COMPADRE study, may increase precision into their impact on cognitive health in this at-risk, underrepresented, population; reduce cultural bias in measurement and outcomes; and facilitate targeted interventions to promote healthy aging.

